# A Bioinformatic Method For Identifying Group II Introns In Organella Genomes

**DOI:** 10.3389/fgene.2019.01135

**Published:** 2019-11-14

**Authors:** Igor Titov, Nikolay Kobalo, Denis Vorobyev, Alexander Kulikov

**Affiliations:** ^1^The Laboratory of Molecular-Genetics Systems, the Federal Research Center Institute of Cytology and Genetics, Novosibirsk, Russia; ^2^The Laboratory of Computational Problems of Geophysics, the Institute of Computational Mathematics and Mathematical Geophysics, Novosibirsk, Russia; ^3^INSERM U981, Gustave Roussy Cancer Center, Villejuif, France

**Keywords:** group II introns, ribonucleic acid, secondary structure, ribozyme, mobile element

## Introduction

Being one of the most successful mobile elements, group II introns are present in all the three domains of life. It is commonly believed that group II introns played an important role in the emergence of eukaryotic retroelements and splicing (Novikova and Belfort, 2017). In biotechnology, group II introns have a promising application as specific gene-targeting vectors (Song et al., 2015).

Group II intron sequences possess weak similarity with each other and at the same time fold into a common (although variable) structure, which provides them with ribozyme activity. The secondary structure of group II introns consists of six distinctive domains, DI-VI. The first of them is organized into a complex structure with a largest number of stems and plays a role of a scaffold on which the whole structure is assembled. The remaining domains have fewer structural elements, although domain V is conserved in sequence and contains the ribozyme’s active site, and domain VI has structural conservation for the branched A motif.

The pioneering collection of group II introns sequences contains a small number of eukaryotic group II introns with several individual structures (Dai et al., 2003). The largest collection containing group II introns, the Rfam, relies on the presence of only DV-VI domains (Kalvary et al., 2018) and on the capability to form secondary structure in the DI domain position and may contain false-positives. To reduce false positive group II introns requires explicit secondary structure models of all domains as well as EBS1-IBS1, EBS2-IBS2 and other tertiary interactions.

This study aims to provide a computational approach to identify group II introns in organellar genomes we applied the structure computation, comparative sequence and structure analysis and the descriptor-based technique that identifies the target structure.

### Value of the Data

1. Group II introns are widespread mobile elements found in bacteria, archaea, mitochondria and chloroplasts that are evolutionarily related to the emergence of splicing and eukaryotic retroelements and are used as a tool in the genome engineering and gene expression control.
2. It would be beneficial to have an automated algorithm that would accurately locate group II introns in organellar genomes, because there is no exhaustive collection of the introns. Here, we provide a dataset of secondary structures of eukaryotic group II introns, supplying them with the data on those tertiary interactions and well-conserved reverse transcriptase (RT)-motifs that we found.
3. These data will be useful for analyzing the complexity of group II intron folding and their evolutionary relationship to eukaryotic retroelements and splicing, for identifying novel group II introns and for TargeTron designing in gene engineering.

## Materials and Methods

To search for structural homologs within RNA sequences, we used the descriptor-based RScan program (http://www.softberry.com/freedownloadhelp/rna/rscan/rscan.all.html). To search for sequence homologs we used NCBI BLAST web server (Madden, 2002). To filter structures by stability we used RNAeval (Lorenz et al., 2011): only those structures that met the criterion Z-score < −1.48 passed the filter. We calculated structures similarity using RNAdistance program of the ViennaRNA Package (Lorenz et al., 2011). As a negative dataset, we generated random 5,000-nt long sequences of the same dinucleotide composition.

Our training set of eukaryotic group II introns structures consisted of eight structures (*Arabidopsis thaliana* nad1.I4, *Porphyra purpurea* LSU.I1 and I2, *Pylaiella littoralis* cox1.I1, I2, and I3, *P. littoralis* LSU.I1 and I2) extracted from the papers (Fontaine et al., 1997; Burger et al., 1999; Sultan et al., 2016; Chan et al., 2018), 10 structures (*Podospora anserina* ND5.I4, cox1.I1 and I4, *Saccharomyces cerevisiae* cox1.I1 and I2, *Schizosaccharomyces pombe* cox1.I1 and cox2.I1, *Venturia inaequalis* cob1.I1, *Glycine max* nad1.I4, *Allomyces macrogynus* cox1.I3) provided by the reviewer 1 and 7 *Marchantia_polymorpha* structures (cob1.I3, cox1.I1 and I2, cox2.I2, atp9.I1, atpA.I2, SSU.I1) from the collection of (Dai et al., 2003).

For all these introns we built topologically distinctive structural models of their DI domains. Each model corresponded to its unique set of stems and started with the 5’ splice site sequence GUGCG, UUGCG, or GGGCG. By varying the lengths of loops and stems within the models and prohibiting non-canonical base pairs (except G-U pair) we searched for structural homologs in eukaryotic sequences hosted in the group II intron database (Dai et al., 2003). Then we filtered the results by the stability criterion and by the presence of interactions EBS1-IBS1, EBS2-IBS2, κ and ε which are necessary for group II intron functioning. In this way, we expanded our training set by two more sequences, *Neurospora crassa* cox1.I1 and *S. pombe* EF2 cob1.I1.

After that, we completed the structures description of group II introns by building the DII–DVI domains models for the training set. Our final training set consisted of 27 sequences, of which 11 included YADD motif and 10 were ORF-less.

Next, we repeated the search for DI structures, but now on the 10,130 eukaryotic sequences of the Rfam database of group II introns (http://rfam.xfam.org/family/RF00029#; note that this dataset contains many duplicate sequences), and then removed the duplicate sequences. Thus, we obtained the thermodynamically stable group II intron-like structures, at the same time clearly losing those group II introns, in which DI domain acquired or lost any stems compared to the DI domain of the training set.

Finally, we built the models for DII–DVI domains using comparative analysis of homologous sequences and the domain models developed for the training set.

Among the training and predicted introns we searched for the YADD sequence (RT active site) and the conserved motifs of RT. We called the RT motif conserved if its similarity to any of the aligned RT-motifs from *Enterococcus faecium*, *Geobacter* sp. *M18*, and *Enterococcus casseliflavus* [the top three sequences in Figure 5 in the paper (Zimmerly and Wu, 2014)] exceeded 70%.

### Data

All the group II introns found, as most of the training sequences, belong to either IIA or IIB canonical model of DI domain (Dai et al., 2008; see also **Supplementary Figure 1**. where small inner loops are not shown). These two DI domain models showed very different false positives, which we evaluated taking into account the tertiary structure motifs and the consensus of the 5’ splicing site. The canonical DI domain model of IIA introns was the most relaxed and showed the highest false positive rate, 3.7·10^−6^ per nt. The false positive rate of the canonical DI domain model of IIB introns was estimated as small as 9.5·10^−8^ per nt. Despite that such a high specificity of IIB case could coexist with a low sensitivity, we found three such correct IIB introns.

**Figure 1:**
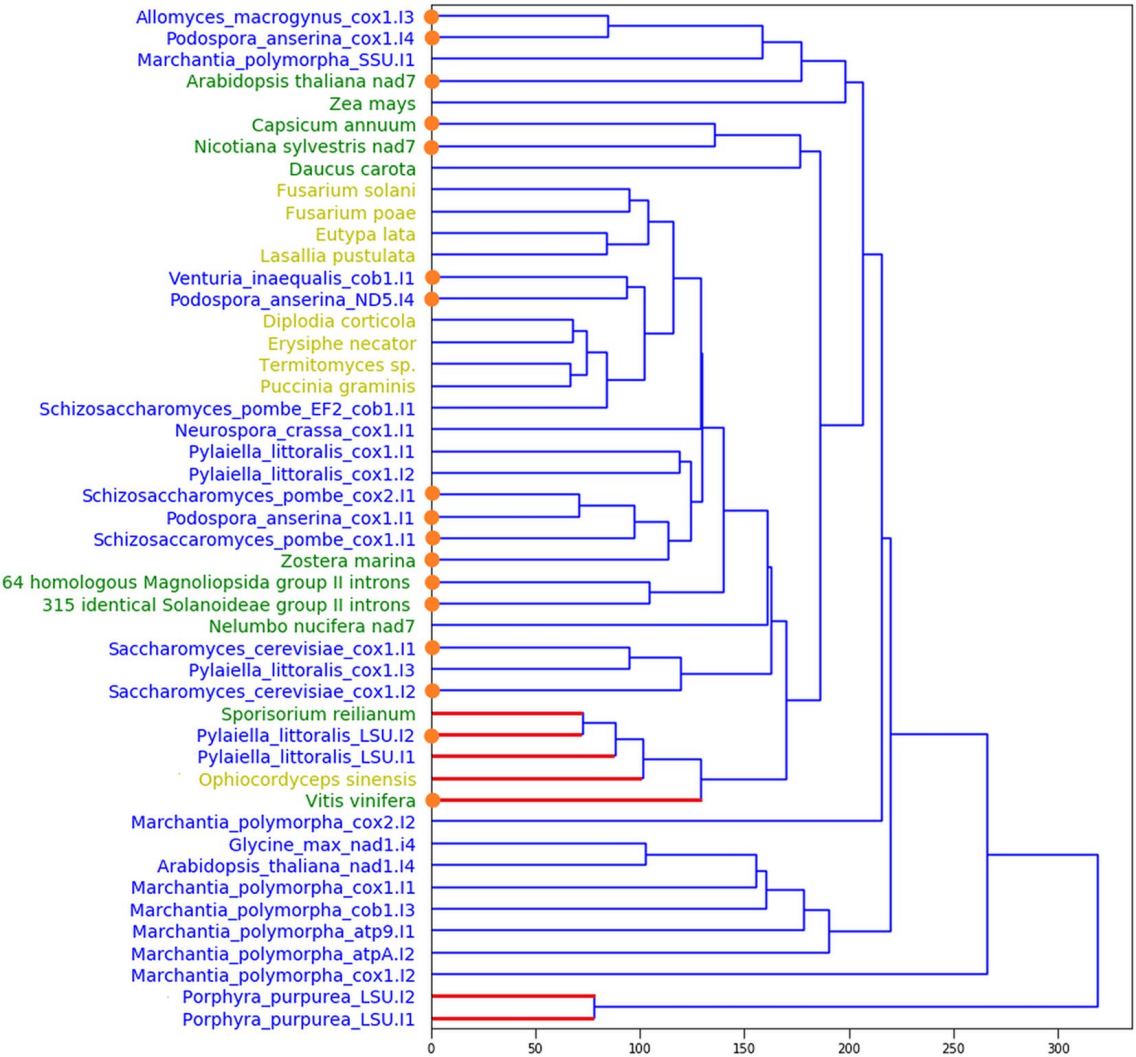
Unweighted pair group method with arithmetic mean tree for DI domain structures of the training and the predicted sets. Blue: the training set; yellow (green): the predicted sequences with YADD (no YADD) motif, correspondingly. Blue (red) edges mark IIA (IIB) introns, correspondingly. By orange dots we show the short (ORF-less) introns.

**Figure 2:**
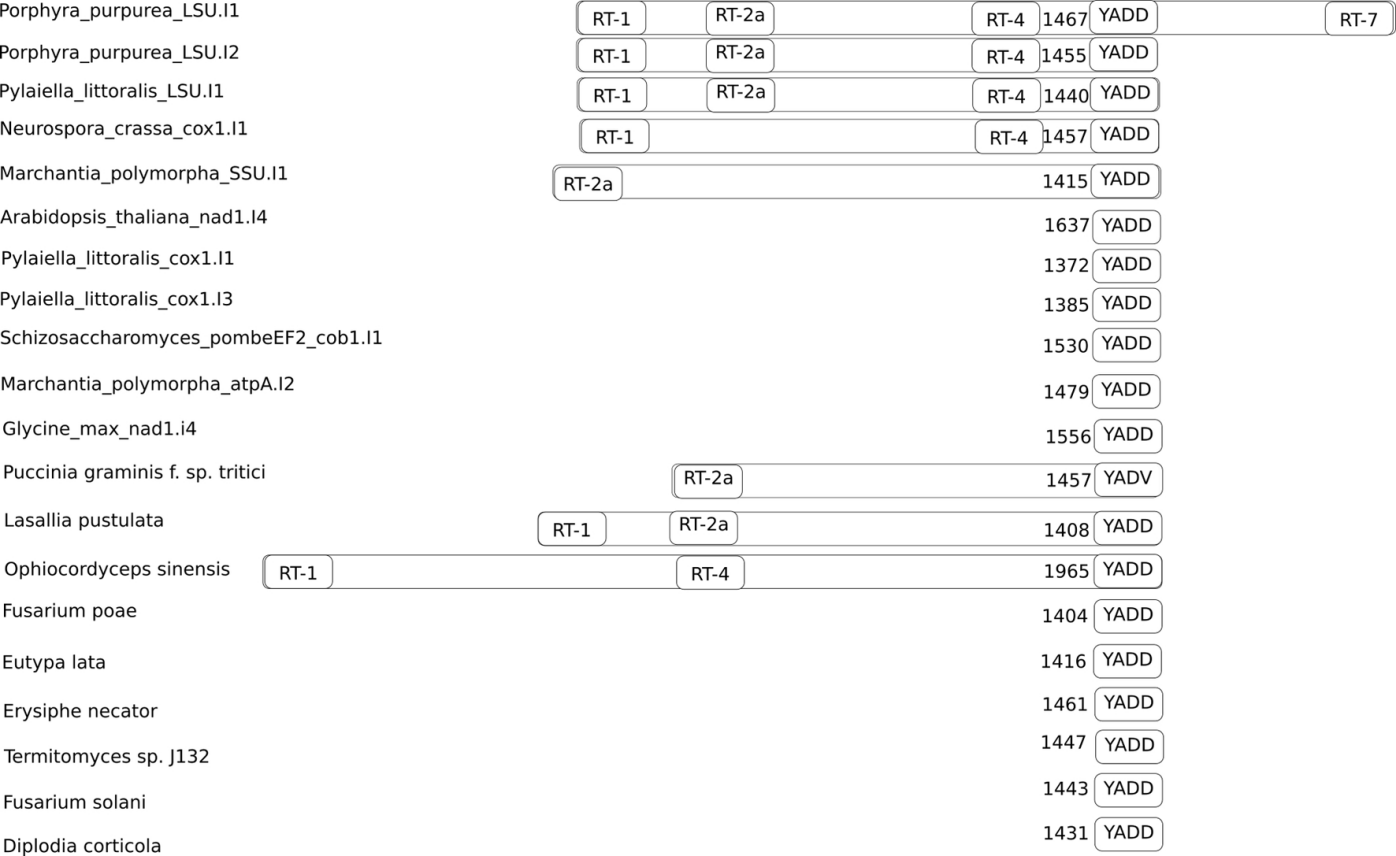
Positions of conservative reverse transcriptase motifs in the training and the predicted sets of group II introns. The number of nucleotides between the YADD motif and the beginning of the intron is shown to the left of the YADD motif. The YADV motif is shown because it can be obtained from YADD by single nucleotide substitution.

In total, we found 397 sequences that satisfied our structural models of DI domains of eukaryotic group II introns. The unweighted pair group method with arithmetic mean tree of their DI domain structural similarity is shown on **Figure 1**. We divided these 397 sequences into three groups. The first group (shown yellow on **Figure 1**) consisted of nine fungi group II introns, which contained, albeit in smaller quantity than in the training set, conserved, and correctly positioned RT-motifs (**Figure 2**). All of these candidates included the YADD motif, had a GUGCG sequence of the 5’ splicing site, seven of them had β-β’ pseudoknots and eight of them were IIA-type. The second group of nine sequences, one fungi and eight plants, had neither YADD nor β-β’ and included five ORF-less introns and two IIB sequences.

The largest, third group consisted of 379 homologous IIA ORF-less *Magnoliopsida* sequences that also had neither YADD nor β-β’, but held a rare UUGCG 5’ splicing site. Inside this group, 64 sequences differed from each other mainly by approximately 150 nt deletion in the DIV domain, indicating a difference in their pathways of DIV degeneration. The 90% homology extends to about 2,500 nt upstream and 1,500 nt downstream from these introns. Despite the similarity of the host genes of these introns, these genes are annotated ambiguously, if annotated. The other 315 group II introns were identical *Solanoideae* sequences in the conserved region, the 90% similarity of which extends to about 20K nt upstream and 3K nt downstream from the intron. The vast majority of this subgroup is missing from the Rfam Database but is detected using the BLAST search, which suggests that many group II introns are still not annotated. It is noteworthy that the third group contained at least 16 nuclear sequences, although the training set consisted only of mitochondrial introns. The information on the sizes of the sets and the characteristics of introns are given in **Supplementary Table 1**.

Interestingly, we have observed that bacterial DI domains can often also fold into the eukaryotic structures, although typically with a lower structure energy. In the group II intron database (Dai et al., 2003) we found 81 such bacterial group II introns, whose DI domains can be folded into eukaryotic DI structures that meet all our criteria for stability and tertiary interactions. These 81 introns could be the closest relatives of the ancestral introns that migrated from bacteria to organelles.

## Data Availability Statement

The sequences and structures of the training set in dot-bracket notation and their graphical images were uploaded to https://figshare.com/articles/Secondary_structures_of_the_training_set_of_eukatyotic_group_II_introns/9034184. The predicted set with the YADD motif can be downloaded from https://figshare.com/articles/secondary_structures_of_eukaryotic_group_II_introns_rfam_set_yadd_/9117092. The predicted set with no YADD motif can be found at https://figshare.com/articles/secondary_structures_of_eukaryotic_group_II_introns_rfam_set_no_yadd_/9117095. The predicted set of homologous IIA ORF-less *Magnoliopsida* sequences and their structures can be downloaded from https://figshare.com/articles/secondary_structures_of_eukaryotic_group_II_introns_rfam_set_no_yadd_homologous_/9930017. The predicted set of identical *Solanoideae* sequences and their structures were uploaded to https://figshare.com/articles/secondary_structures_of_eukaryotic_group_II_introns_rfam_set_multicopy_/9930110. Each sequence was also provided with its splicing site sequence and EBS1-IBS1, EBS2-IBS2, α-α’ and β-β’ pseudoknots if found.

## Author Contributions

IT designed and coordinated the study, carried out bioinformatics analysis, and wrote the manuscript. NK carried out the programming, bioinformatics, and statistical calculations. DV carried out the programming and bioinformatics analysis. AK carried out the programming. All authors have read and approved the final manuscript.

## Funding

The work of IT has been supported by the Budget project 0324-2019-0040.

## Conflict of Interest

The authors declare that the research was conducted in the absence of any commercial or financial relationships that could be construed as a potential conflict of interest.
