# Supplementary figures and images for: A Bioinformatic Method For Identifying Group II Introns In Organella Genomes
Source: Front Genet. 2019 Nov 14;10:1135. doi: 10.3389/fgene.2019.01135 (PMC6867995; doi:10.3389/fgene.2019.01135)

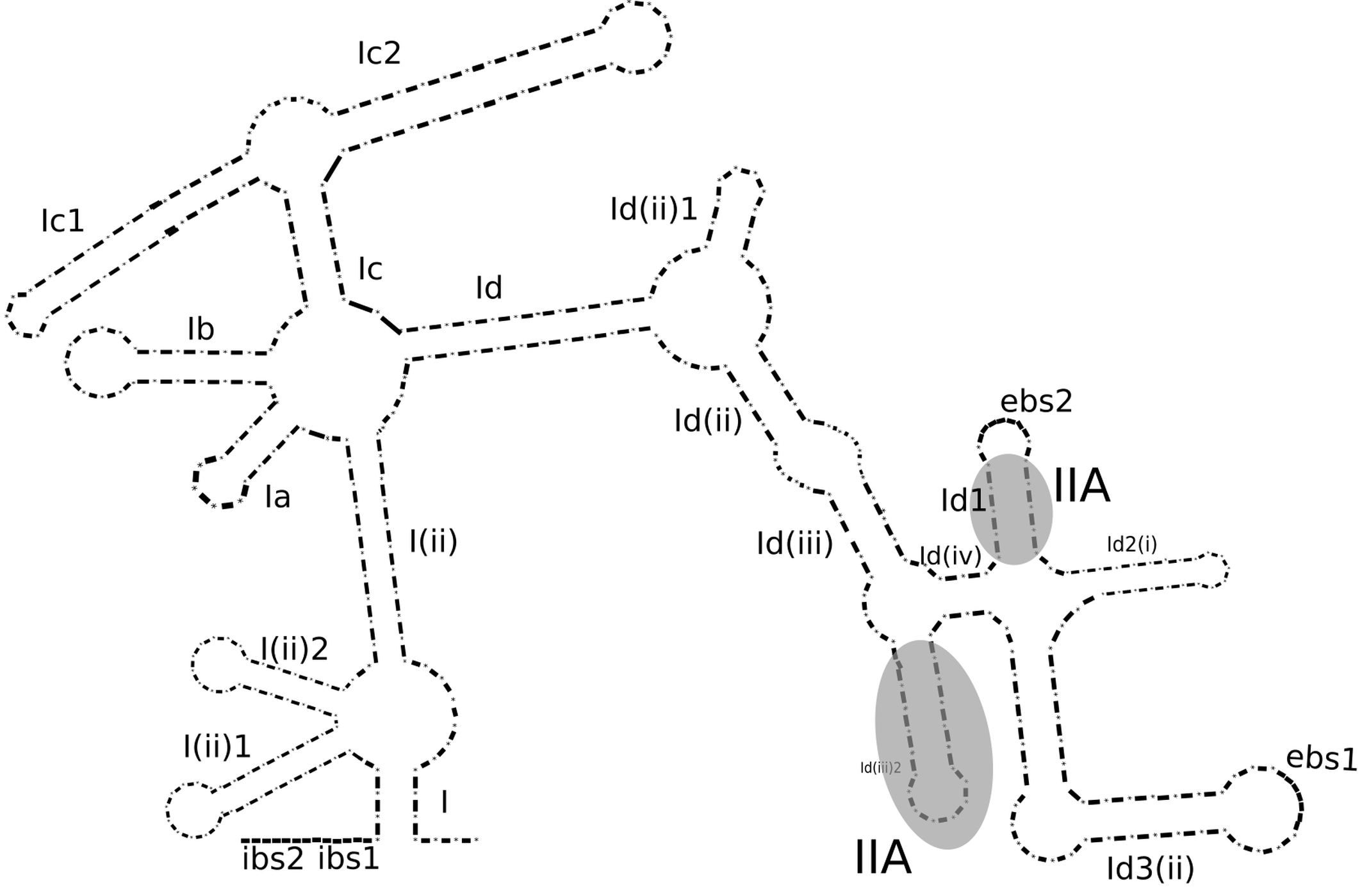

Supplement: Supplementary file 1 [file Image_1.jpeg]
