# Supplementary material for: A Bioinformatic Method For Identifying Group II Introns In Organella Genomes
Source: Front Genet. 2019 Nov 14;10:1135. doi: 10.3389/fgene.2019.01135 (PMC6867995; doi:10.3389/fgene.2019.01135)
Supplement: Supplementary file 2 [file Table_1.doc]

|  | sample_size | yadd | alpha | beta | nuclear | mitochondrial | chloroplast | plastid | GUGCG | UUGCG | GGGCG |
| --- | --- | --- | --- | --- | --- | --- | --- | --- | --- | --- | --- |
| training long | 17 | 11 | 14 | 6 | 0 | 17 | 0 | 0 | 14 | 1 | 1 |
| training short | 10 | 0 | 9 | 4 | 0 | 10 | 0 | 0 | 10 | 0 | 0 |
| predicted long | 13 | 9 | 10 | 7 | 2 | 5 | 0 | 0 | 11 | 2 | 0 |
| predicted short | 5 | 0 | 4 | 0 | 0 | 3 | 0 | 0 | 5 | 0 | 0 |
| Magnoliopsida homologous | 64 | 0 | 64 | 0 | 14 | 0 | 12 | 0 | 0 | 64 | 0 |
| Solanoideae identical | 315 | 0 | 315 | 315 | 2 | 2 | 115 | 196 | 0 | 315 | 0 |
